# Flavonoids inhibit cell proliferation and induce apoptosis and autophagy through downregulation of PI3Kγ mediated PI3K/AKT/mTOR/p70S6K/ULK signaling pathway in human breast cancer cells

**DOI:** 10.1038/s41598-018-29308-7

**Published:** 2018-07-26

**Authors:** Hong-Wei Zhang, Jin-Jiao Hu, Ruo-Qiu Fu, Xin Liu, Yan-Hao Zhang, Jing Li, Lei Liu, Yu-Nong Li, Qin Deng, Qing-Song Luo, Qin Ouyang, Ning Gao

**Affiliations:** College of Pharmacy, 3rd Military Medical University, Chongqing, 400038 China

## Abstract

Anticancer activities of flavonoids derived from *Tephroseris kirilowii* (Turcz.) Holub. were evaluated in human cancer cells. We isolated and identified, for the first time, eight flavonoids from *T*. *kirilowii* and found that three of them (IH: isorhamnetin, GN: genkwanin, and Aca: acacetin) inhibited cell proliferation in a variety of human cancer cell lines. These active flavonoids caused cell cycle arrest at G2/M phase and induced apoptosis and autophagy in human breast cancer cells. Molecular docking revealed that these flavonoids dock in the ATP binding pocket of PI3Kγ. Importantly, treatment with these flavonoids decreased the levels of PI3Kγ-p110, phospho-PI3K, phospho-AKT, phospho-mTOR, phospho-p70S6K, and phospho-ULK. Pretreatment with PI3Kγ specific inhibitor AS605240 potentiated flavonoids-mediated inactivation of AKT, mTOR, p70S6K, ULK, and apoptosis. Taken together, these findings represent a novel mechanism by which downregulation of PI3Kγ-p110 and consequent interruption of PI3K/AKT/mTOR/p70S6K/ULK signaling pathway might play a critical functional role in these flavonoids-induced cell cycle arrest at G2/M phase, apoptosis, and autophagy. Our studies provide novel insights into the anticancer activities of selected flavonoids and their potential uses in anticancer therapy.

## Introduction

Traditional Chinese medicines have been recently recognized as a new source of anticancer drugs and neoadjuvant chemotherapy to enhance the efficacy of chemotherapy and to alleviate the side effects of cancer chemotherapy^1,2^. However, the mechanisms of actions are still largely unknown. Flavonoids are a group of more than 4000 polyphenolic compounds that occur naturally in a variety of plant origin^3^. A growing number of studies indicate that flavonoids or flavonoid derivatives play critical roles in cancer chemoprevention and chemotherapy. A number of epidemiological studies indicate that high flavonoid intake may be correlated with a decreased risk of cancer, and provide evidence for the protective roles of flavonoids against cancer^4,5^. *In vitro* studies indicate that anticancer activities of flavonoids may be related to inhibiting cell proliferation, adhesion, and invasion, inducing cell differentiation, cell cycle arrest, and apoptosis, etc.^6,7^. *In vivo* studies demonstrate that flavonoids could inhibit carcinogenesis by affecting the molecular events in the initiation, promotion, and progression stages^8^. The clinical trials of flavonoids in human have been exploited to achieve cancer preventive or therapeutic effects^9^. Based on these results, flavonoids could be developed as promising agents for cancer chemoprevention and chemotherapy.

*Tephroseris kirilowii Turcz*. *Holub* (Compositae) is a perennial herb widely distributed in China^10^. The whole plant of *T*. *kirilowii* exhibit a wide range of biological activities against many types of diseases such as urethral infection, oedema, eczema, scabies, vaginal trichomoniasis, and leukaemia in Chinese-folk medicine^11–13^. The main constituents of *T*. *kirilowii* are alkaloids and flavonoids. Recently, natural compounds from flavonoids have been found to exhibit anti-cancer effects through multiple molecular mechanisms that involve the modulation of apoptosis, cell cycle arrest and autophagy^14–16^. However, the types of flavonoids in *T*. *kirilowii* have not been characterized, nor have the mechanisms of flavonoids-mediated anticancer activities been elucidated in depth. The purpose of the present study is to isolate and characterize the structures of flavonoids from *T*. *kirilowii*, to evaluate the effects of flavonoids on anticancer activities, and to elucidate the molecular mechanisms of flavonoids-mediated anticancer activities. In this study, we isolated and identified, for the first time, eight flavonoids from *T*. *kirilowii*: isorhamnetin, isorhamnetin-3- glucoside, kaempferol, apigenin, acacetin, chrysin, 7,8-dihydroxyflavanone, and genkwanin. Using bioactivity-guided screening of selected flavonoid compounds isolated from *T*. *kirilowii*, we found that three compounds among these flavonoids exhibited anticancer activities. We provide the evidence that isorhamnetin, genkwanin, and acacetin inhibited cell proliferation in human cancer cells through cell cycle arrest at G2/M phase and induction of apoptotic and autophagic cell death. Molecular docking revealed that these flavonoids dock in the ATP binding pocket of PI3Kγ. Mechanistic studies revealed that interruption of the PI3K/Akt/mTOR/ULK signaling pathway plays a critical role in these flavonoids-mediated cell cycle arrest and induction of apoptotic and autophagic cell death via reducing PI3Kγ-p110 expression. Our studies provide novel insights into the anticancer activities of selected flavonoids and their potential uses in anticancer therapy.

## Materials and Methods

### Isolation, purification and identification of flavonoids from *Tephroseris kirilowii (Turcz*.)* Holub*

*Tephroseris kirilowii (Turcz*.*) Holub*. was purchased from a vendor (Guoan Chinese Herbal Medicine Co. Ltd. Zhengzhou, Henan, China). *T*. *kirilowii* was extracted with 70% MeOH for 3 days at room temperature to obtain a crude extract. This extract was suspended in 10% aqueous MeOH and partitioned between hexane, CHCl_3,_ EtOAc, and BuOH to obtain the corresponding dried extracts. The EtOAc extract was subjected to silica gel column chromatography using CHCl_3_-MeOH solvent systems of increasing polarity to afford fractions A to C. Fraction A-C purified respectively by SephadexLH-20 CC (CHCl_3_/MeOH, 10:90) to yield eight flavonoids. These eight flavonoids were further purified by high-performance liquid chromatography (HPLC).

The structures of flavonoids were identified by spectroscopic analyses including MS and NMR (nuclear magnetic resonance).

### Chemicals and antibodies

AS-605240 (S1410) and nocodazole were purchased from Selleck Chemicals (Shanghai, CA). Antibodies against PI3Kγ (5405 T), phospho-Akt (Ser473) (4051), Akt (2920), phospho-mTOR (Ser2448) (2971 L), mTOR (2972), phospho-ULK1 (Ser757) (6888), ULK1 (8054S), phospho-p70S6K1 (Thr389) (9204), cleaved caspase-3 (9661S), pro-caspase-3 (9668S) and GAPDH (5174) were from Cell Signaling Technology (Beverly, MA); XIAP (610716) and Mcl-1 (559027) were from BD Biosciences; PARP was from Epitomics (32561).

### Cell culture

MDA-MB-231, MCF-7, A549, SMMC-7721, Eca109, HEB and MCF-10A cells were provided by the American Type Culture Collection (ATCC, Manassas, VA). Cells were cultured in DMEM, RPMI1640 and MEBM medium contained 10% fetal bovine serum (FBS) and antibiotics at 37 °C in a humidified atmosphere and 5% CO_2_ in air.

### Cell viability (MTT) assay

Cells (5 × 10^3^) were seeded in each well of 96-well plates and treated as indicated experimental conditions for 24 h. 20 μl MTT (5 mg/ml) was added per well and incubated at 37 °C for 4 h. MTT assay was performed according to the manufacturer’s instruction. The cell viabilities were normalized to the control group. The IC_50_ values were calculated by using linear-regression analysis.

### Apoptosis assay

Cells were stained with annexin V-FITC and PI to evaluate apoptosis by flow cytometry according to the manufacturer’s instructions (BD Biosciences PharMingen). In brief, 1 × 10^6^ cells were washed twice with phosphate-buffered saline (PBS) and stained with 2 μl of Annexin V-FITC and 5 μl of PI (50 μg/ml) in 1× binding buffer (10 mM of HEPES, pH 7.4, 140 mM of NaOH, and 2.5 mM of CaCl_2_) for 15 min at room temperature in the dark. Quantification of apoptotic cells was performed by flow cytometry using a FACScan cytofluorometer (BD Biosciences). Both early (Annexin V-positive, PI-negative) and late (Annexin V-positive and PI-positive) apoptotic cells were included in the cell death determinations.

### Cell cycle analysis

Cells were harvested, washed twice with phosphate-buffered saline (PBS) and incubated with nuclei staining buffer (0.1% Triton X-100, 3.8 mM sodium citrate, RNase B (7 kU/ml), and PI solution (50 μg/ml) for 2 h. The cell cycle distribution was measured by using the Becton-Dickinson FACScan cytofluorometer (Mansfield, MA, USA).

### Western blots

Cells were processed for western blotting as described previously^17^. In brief, 20–40 μg of sample proteins were separated using SDS-PAGE and transferred to PVDF membranes (Bio-Rad, 162–0177). The membrane was probed with antibodies as indicated. The membrane was then incubated horseradish peroxidase-conjugated secondary antibodies (Kirkegaard and Perry Laboratories, Gaithersburg, MD), and the signal was detected using a chemiluminescence reagent kit (Amersham Pharmacia, Piscataway, NJ).

### Molecular docking study

The binding modes of three flavonoids (IH, GN, and Aca) with kinases were predicted by Surflex-Dock in SYBYL2.0. The crystal structures of kinases were retrieved from the Protein Data Bank (http://www.pdb.org/pdb/) and prepared by SYBYL-X 2.0 (including residue repair and energy minimization). In the process of molecular docking, we extract the original ligands out of the protein and generate banding pocket. Subsequently, original ligands, Aca, IH, and GN were docked into the pocket respectively.

### PI3K kinase assay

ADP-Glo luminescent assay for PI3Kγ was performed according to the standard protocols of Promega. In brief, the kinase reaction was done in 96-well plate. Each well was loaded with test items and reaction buffer containing PI substrate and the PI3K proteins were then added. The reaction was started by the addition of PIP2 and ATP prepared in the reaction buffer and ran for 60 min and subsequently terminated by the addition of ADP-Glo reagent, then read the plate for luminescence detection.

### Statistical analysis

Statistical analysis was performed with SPSS 20 software (SPSS, Chicago, Illinois, USA). Data were represented as means ± SD. For comparison between two data sets, a Student’s t test was used. For analysis of three or more sets of data, ANOVA was used. *P < 0.05, **P < 0.01 were considered statistically significant.

## Results

### Anticancer activity screening of flavonoid compounds from *T. kirilowii*

**Figure 1:**
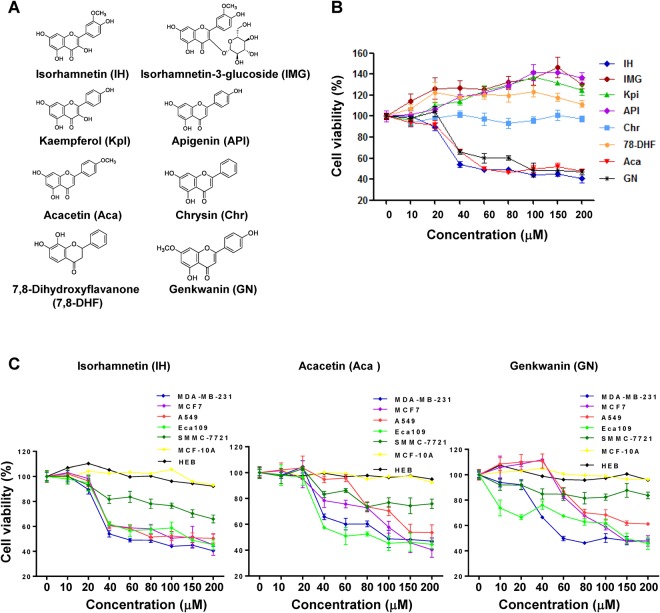
IH, GN and Aca inhibit cell proliferation in multiple cancer cell lines. (**A**) Structure of IH, IMG, Kpl, API, Aca, Chr, 7,8-DHF and GN. (**B**) MDA-MB-231 cells were treated with increasing doses of flavonoids for 24 h, and MTT assays were performed to assess cell proliferation. (**C**) Multiple cancer cell lines were treated with increasing doses of IH, GN and Aca for 24 h, and cell proliferation were measured by MTT assay. IC_50_ values were presented as mean ± SD for three separate experiments.

Eight flavonoid compounds were isolated for the first time from *T*. *kirilowii*. Their structures were identified by means of spectroscopic analyses as isorhamnetin (IH), isorhamnetin-3-glucoside (IMG), kaempferol (Kpl), apigenin (API), acacetin (Aca), chrysin (Chr), 7,8-dihydroxyflavanone (7,8-DHF), and genkwanin (GN) (Fig. 1A). To evaluate the effects of these flavonoids on the growth of human cancer cells, the growth inhibitory potential of these compounds was determined in human breast cancer MDA-MB-231 cells. We found that the cell viabilities were decreased in a dose-dependent manner in cells treated with IH, GN, and Aca (Fig. 1B). The IC_50_ values of IH, GN, and Aca for inhibition of cell proliferation in MDA-MB-231 cells were 55.51 μM, 58.54 μM, and 82.75 μM, respectively (Table 1). In contrast, the cell viabilities were slightly increased or not changed in cells treated with other five flavonoids with IC_50_ values greater than 200 μM (Fig. 1B, Table 1).

**Table 1:** IC50 of Flavonoids-inhibited cell proliferation in a variety of cancer.

Compounds	MDA-MB-231	MCF-7	SMMC-7721	A549	Eca109
IMG	>200	>200	>200	>200	>200
IH	55.51 ± 5.22	98.76 ± 4.73	>200	148.44 ± 10.34	103.71 ± 6.72
Kpl	>200	>200	>200	>200	>200
API	>200	>200	>200	>200	>200
Chr	>200	>200	>200	>200	>200
GN	58.54 ± 3.67	101.40 ± 3.64	>200	120.77 ± 4.24	98.2 ± 3.23
7,8-DHF	>200	>200	>200	>200	>200
Aca	82.75 ± 4.35	103.91 ± 4.65	>200	157.40 ± 7.54	54.7 ± 5.52

To determine whether the inhibition of cell proliferation mediated by IH, GN, and Aca was restricted to breast cancer cells, parallel studies were performed in a variety of cancer cells including breast cancer MCF-7 cells, hepatocellular carcinoma SMMC-7721 cells, lung adenocarcinoma A549 cells, and esophageal carcinoma Eca109 cells. The inhibitory effects of cell proliferation mediated by three active flavonoids in these cancer cell lines were similar to that in MDA-MB-231 cells, but had little effect on human normal glial cell line HEB and non-tumorigenic epithelial cell line MCF-10A (Fig. 1C).

### IH, GN, and Aca cause cell cycle arrest at G2/M phase

**Figure 2:**
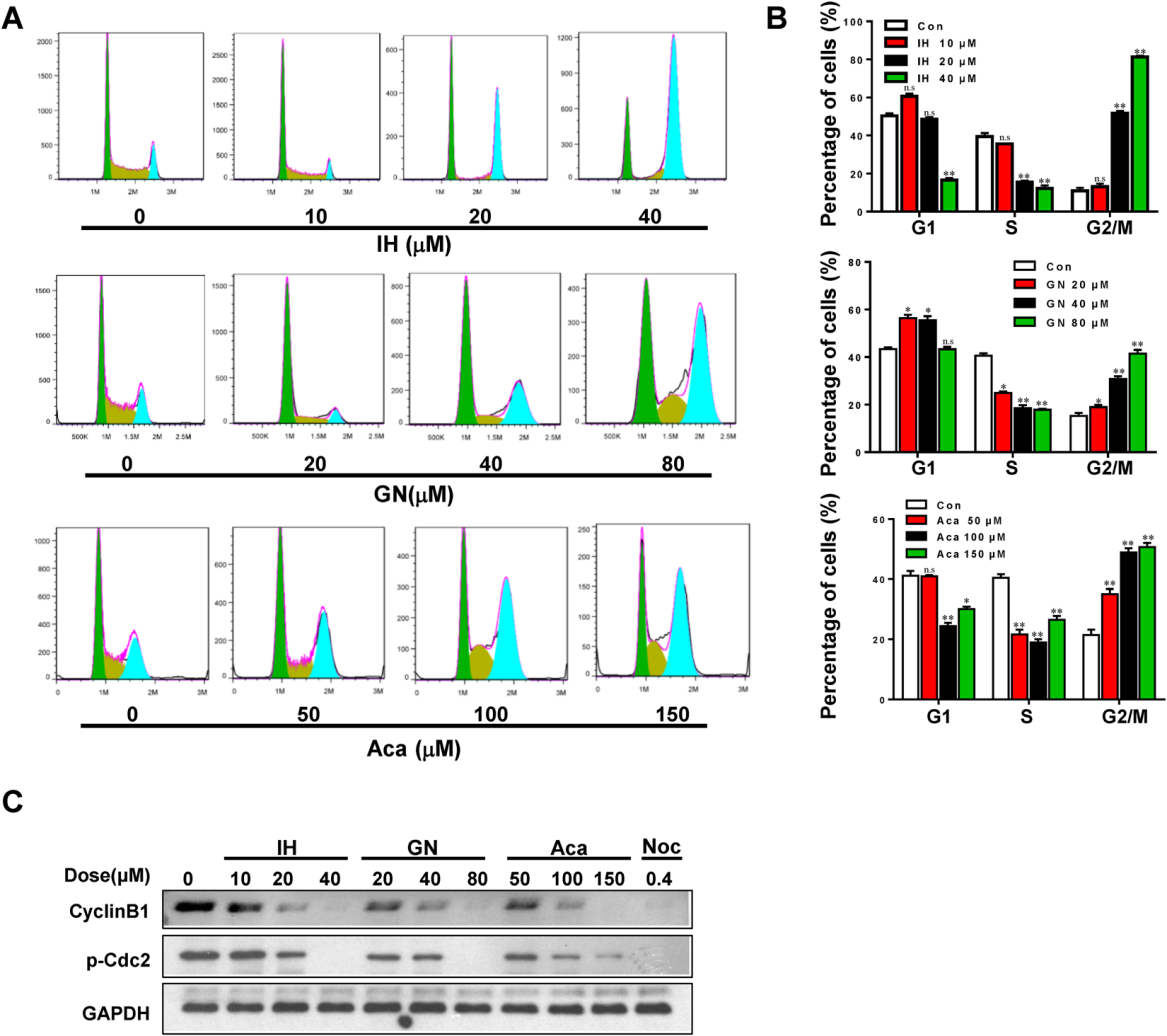
IH, GN and Aca cause cell cycle arrest in MDA-MB-231. MDA-MB-231 cells were treated with IH, GN, Aca at indicated concentrations for 24 h. (**A**,**B**) Cells were stained with cell cycle staining solution and analyzed using a flow cytometer. The percentage of cells in each phase is showed as mean ± S.D. for three independent experiments (^n.s^*P* > 0.05, *P < 0.05 or **P < 0.01 vs. the control). (**C**) Cell lysates were prepared and analyzed by western blotting using antibodies against phospho-Cdc2 and cyclin B1.

To explore the mechanism by which IH, GN, and Aca inhibit cell proliferation, we next investigated the effects of these active flavonoids on cell cycle progression in human breast cancer MDA-MB-231 cells by using flow cytometry assay. The typical histograms of cell cycle distribution in cells treated without or with IH, GN, and Aca for 24 h were shown in Fig. 2A. Treatment with these flavonoids resulted in increase in percentage of cells at G2/M phase and decrease in percentage of cells at G1 and S phase in a dose-dependent manner (Fig. 2B). These results suggest that flavonoids-mediated inhibitory effects of cell proliferation were accompanied by cell cycle arrest at G2/M phase. G2/M cell cycle arrest was also observed in another breast cancer MCF-7 cells-treated with IH, GN, and Aca (Fig. S1).

To explore the mechanism by which these flavonoids induce cell cycle arrest at the G2/M phase, we determined if flavonoids modulate the expression of G2/M cell cycle regulatory molecules using western blotting. Exposure of MDA-MB-231 cells to IH, GN, and Aca resulted in marked decrease in levels of phospho-Cdc2 and cyclin B1 in a dose-dependent manner (Figs 2C, S2). These events were also confirmed in MCF-7 cells-treated with IH, GN, and Aca (Fig. S1). Such findings suggest that repression of Cdc2 and cyclin B1 is likely to be involved in flavonoids-induced G2/M arrest.

**Figure 3:**
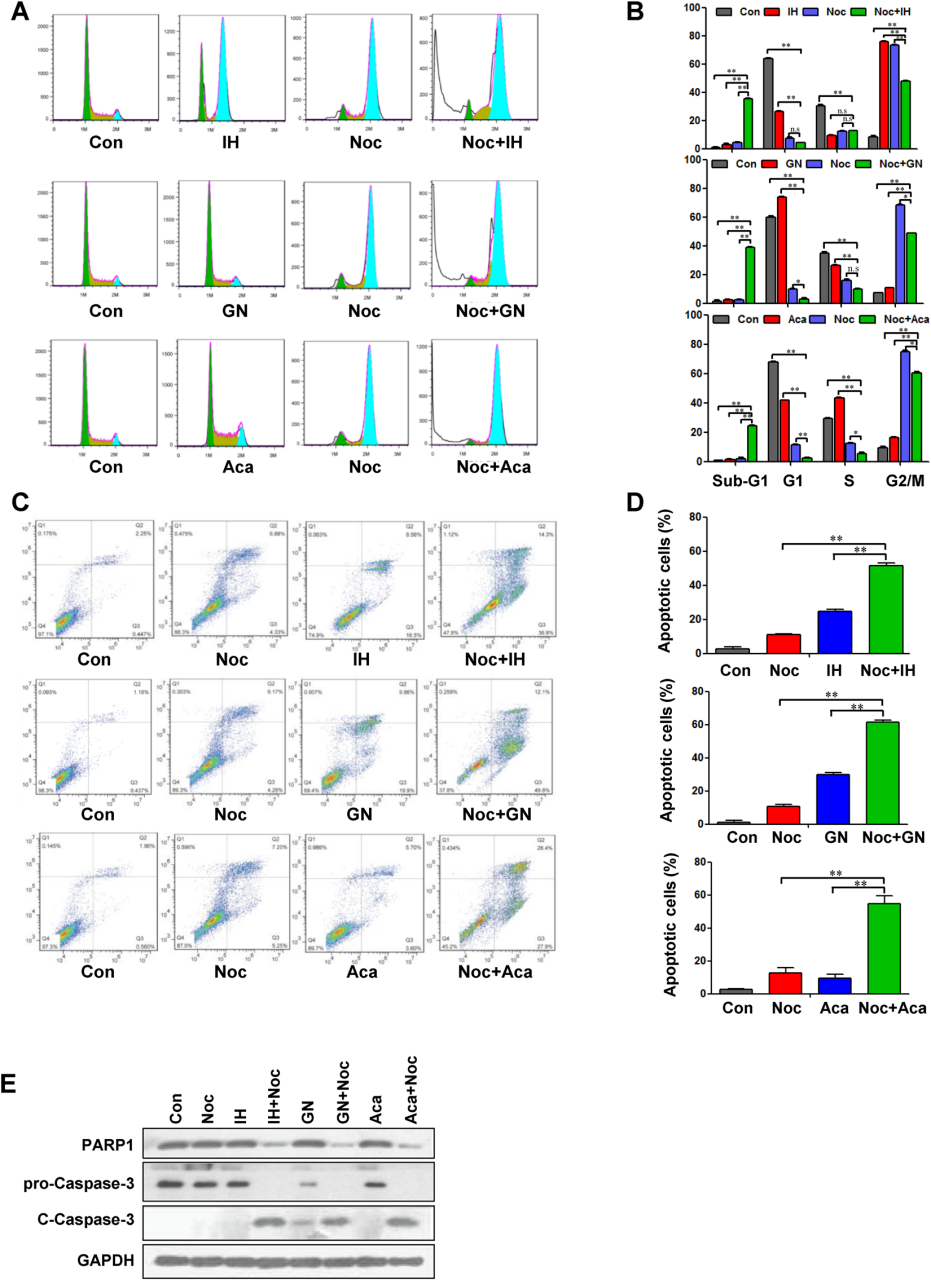
Pretreatment with nocodazole enhance IH, GN and Aca-induced apoptosis in MDA-MB-231 cells. MDA-MB-231 cells were pretreated with 100 ng/ml nocodazole for 12 h, followed by treatment with or without 30 μM IH, 20 μM GN and 100 μM Aca for 24 h. (**A**,**B**) Cells were stained with cell cycle staining solution and analyzed using a flow cytometer. The percentage of cells in each phase is showed as mean ± S.D. for three independent experiments (^n.s^*P* > 0.05, ^*^P < 0.05 or ^**^P < 0.01). (**C**,**D**) Cells were stained with Annexin V/PI, and the percentage of apoptotic cells were determined using flow cytometry. Data was showed as mean ± S.D (^**^P < 0.01) for three independent experiments. (**E**) Cell lysates were prepared and analyzed by western blotting using antibodies against PARP1, pro-caspase-3 and cleaved-caspase 3 (C-Caspase 3).

To determine whether G2/M-arrested cells are susceptible to flavonoids-induced cell death, we examined the effect of IH, GN, and Aca in cells synchronized at the G2/M phase by nocodazole treatment. Treatment of G2/M-synchronized cells with low concentrations of IH, GN, and Aca resulted in a pronounced increase in the proportion of sub-G1 cell population (Fig. 3A,B). Furthermore, treatment of G2/M-synchronized cells with flavonoids also resulted in a marked increase in apoptosis (Figs 3C–E, S3). Such findings suggest that G2/M-arrested cells might be sensitive to undergoing induction of apoptosis-mediated by IH, GN, and Aca.

### IH, GN, and Aca induce apoptosis

**Figure 4:**
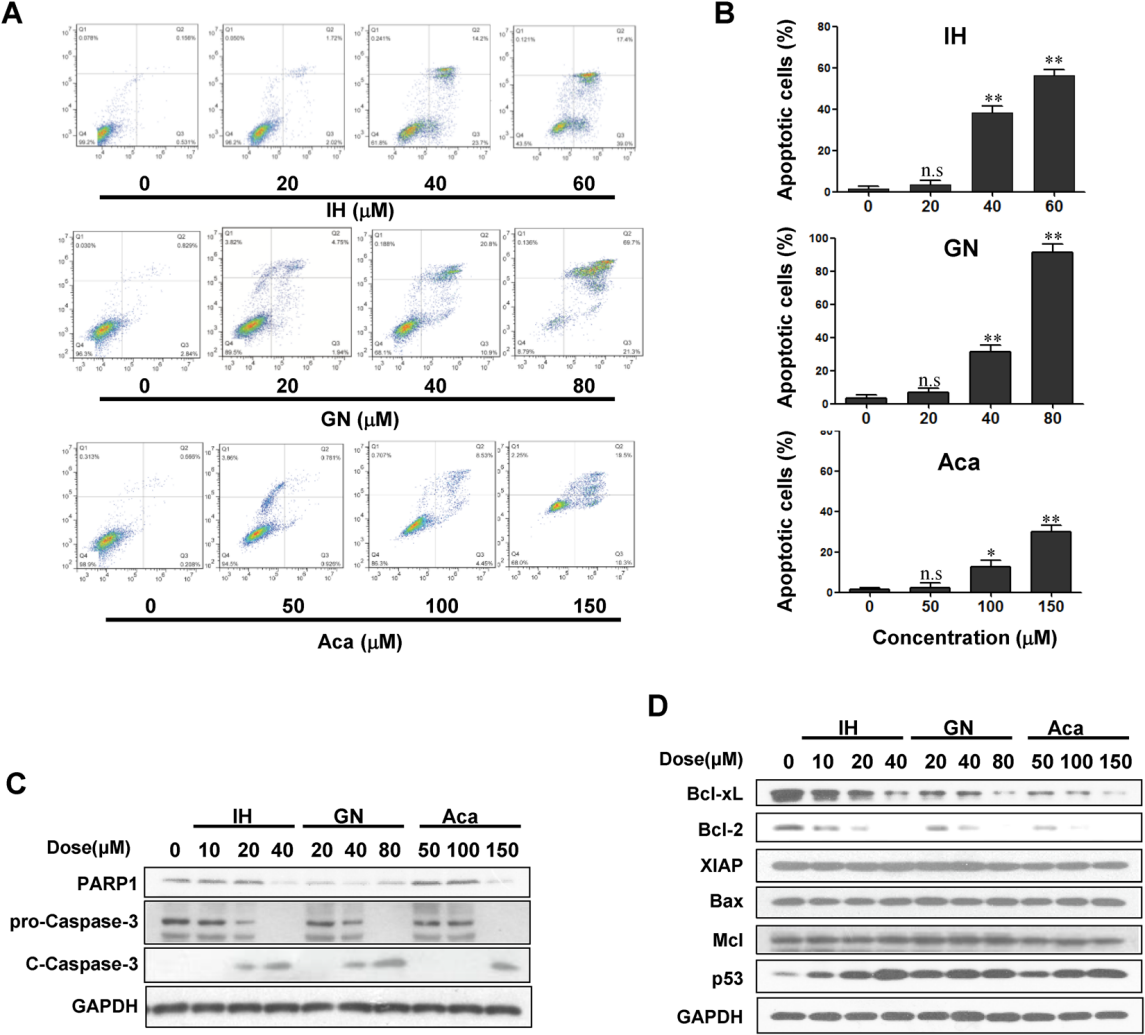
IH, GN and Aca induce apoptosis in MDA-MB-231. MDA-MB-231 cells were treated with IH, GN, Aca at indicated concentrations for 48 h. (**A**,**B**) The percentage of apoptotic cells were determined by flow cytometry using Annexin V/PI staining for three independent experiments. (mean ± SD, ^n.s^*P* > 0.05, ^*^P < 0.05 or ^**^P < 0.01 vs. the control). (**C**,**D**) Cell lysates were prepared and analyzed by western blotting using antibodies against PARP1, pro-caspase-3, cleaved-caspase 3 (C-Caspase 3), Bcl-2, Bcl-xL, p53, Bax, XIAP and Mcl-1.

We next investigated whether IH, GN, and Aca inhibited cell proliferation through induction of apoptosis by flow cytometry assay. Treatment of MDA-MB-231 cells with these flavonoids resulted in increase in apoptosis in a dose-dependent manner (Fig. 4A,B). IH and GN exhibited more potent activities in apoptosis induction than Aca. Consistent with these findings, treating cells with same concentrations of flavonoids resulted in decrease in the expression of PARP1 and increase in levels of cleaved caspase-3 (Figs 4C, S4A). These events were also confirmed in MCF-7-treated with IH, GN, and Aca (Fig. S5).

To gain further insight into the mechanism of flavonoids-induced apoptosis, we examined the expression of Bcl-2 family proteins and the tumor suppressor gene p53 using western blot analysis. Treatment of cells with IH, GN, and Aca resulted in decrease in levels of Bcl-2 and Bcl-xL and increase in levels of p53 (Figs 4D, S4B). In contrast, the expressions of other Bcl-2 family proteins like XIAP, Mcl-1, and Bax remained largely unchanged with treatment of flavonoids (Figs 4D, S4B). Together, these findings suggest that upregulation of p53 and downregulation of Bcl-2 and Bcl-xL could be involved in flavonoids-induced apoptosis in breast cancer cells.

### IH, GN, and Aca induce autophagic cell death

**Figure 5:**
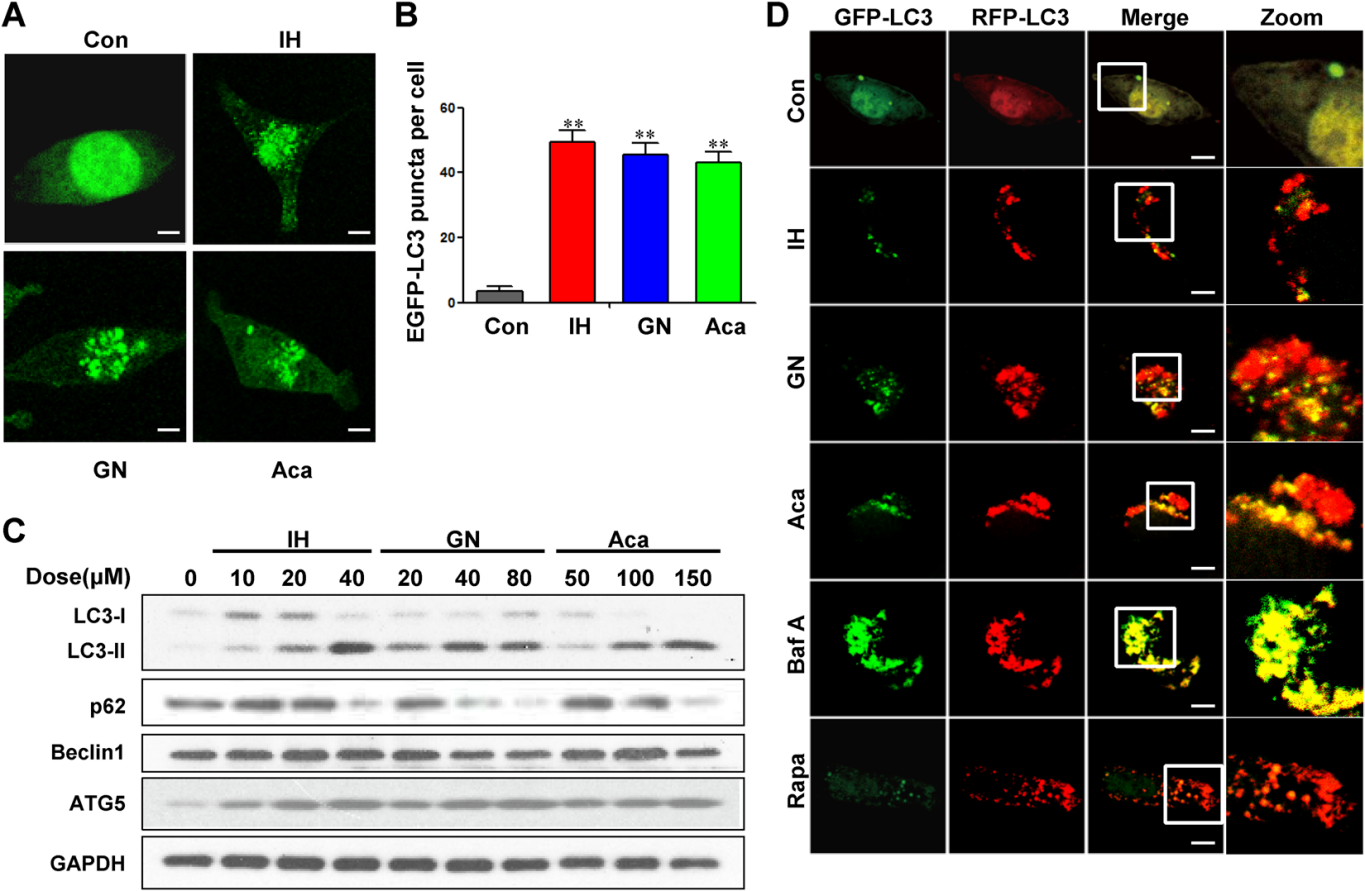
IH, GN and Aca induce autophagy in MDA-MB-231. (**A**,**B**) MDA-MB-231 cells transfected with EGFP-LC3 were treated with 40 μM IH, 20 μM GN and 100 μM Aca for 24 h, the EGFP-LC3 puncta were observed under confocal microscopy; scale bars: 10 μm. Quantification of average EGFP puncta per cell for three independent experiments. Data was presented as mean ± SD (^**^P < 0.01 vs. the control). (**C**) Cells were exposed to indicated concentrations of IH, GN and Aca for 24 h, the expression of autophagy-related proteins (LC3B-II, p62, Beclin1, ATG5) were detected by western blot analysis, GAPDH was used as a loading control. (**D**) Cells were transfected with a tandem reporter construct (tfLC3), and were exposed to 30 μM IH, 20 μM GN, 100 μM Aca, 20 nM Baf and 0.25 μM Rapa as indicated. The colocalization of EGFP and mRFP-LC3 puncta was examined by confocal microscopy. Scale bars: 10 μm.

To verify whether the selected flavonoids were capable of inducing autophagy, we first used MDA-MB-231 cells transiently expressing EGFP-LC3. Autophagosome accumulation can be detected with a confocal laser-scanning microscope. Exposure of cells to IH, GN, and Aca resulted in marked increases in EGFP-LC3 puncta formation in MDA-MB-231 cells (Fig. 5A,B). Western blot analysis showed that treatment of cells with IH, GN, and Aca resulted in a dose-dependent accumulation of LC3-II (Figs 5C, S6). Treatment of cells with IH, GN, and Aca also resulted in a dose-dependent decrease in levels of p62 and increase in levels of ATG5 (Figs 5C, S6). These results suggest that these flavonoids may act as autophagy inducers which promoted p62 degradation by autophagy.

To further examine whether the effects of these flavonoids are due to induced autophagic flux, cells transfected with a tandem reporter construct (tfLC3) were treated with IH, GN, and Aca followed by assessment of EGFP-LC3 and mRFP-LC3 puncta colocalization. EGFP-fluorescence is quenched in acidic environments, whereas mRFP is more stable under acidic conditions^18^. As shown in Fig. 5D, exposure of cells to IH, GN, and Aca caused pronounced formation of LC3 puncta that displayed only red fluorescence intensity, which was similar to that in cells treated with rapamycin (a typical autophagy inducer). In contrast, treatment with bafilomycin A, a vacuolar-type ATPase inhibitor that blocks autophagic degradation, caused pronounced formation of LC3 puncta that displayed both green and red fluorescence intensity producing a yellow overlay. Induction of autophagy was also observed in MCF-7 cells-treated with IH, GN, and Aca (Fig. S7). Thus, these data clearly demonstrate that these flavonoids act as autophagy inducers, which promote autophagic degradation.

### IH, GN, and Aca dock in the ATP binding pocket of PI3Kγ

Molecular docking plays an important role in structure-based drug designing, functional site prediction on protein molecular surfaces, protein ligand docking, etc.^19,20^. A number of evidence revealed that phosphatidylinositol 3-kinase (PI3K)/Akt and mitogen-activated protein kinase (MAPK, including ERK, p38, and JNK) signaling pathways play critical roles in the regulation of cell proliferation, apoptosis, and autophagy^21,22^. To explore the possible interaction between flavonoids and PI3K/Akt or MAPK, molecular docking of three active flavonoids with these signaling pathways was performed. After evaluating the interaction by analyses of the docking scores and binding poses, PI3Kγ was predicted as the most possible target because these flavonoids have similar binding poses as the original ligands in the crystal structure.

**Figure 6:**
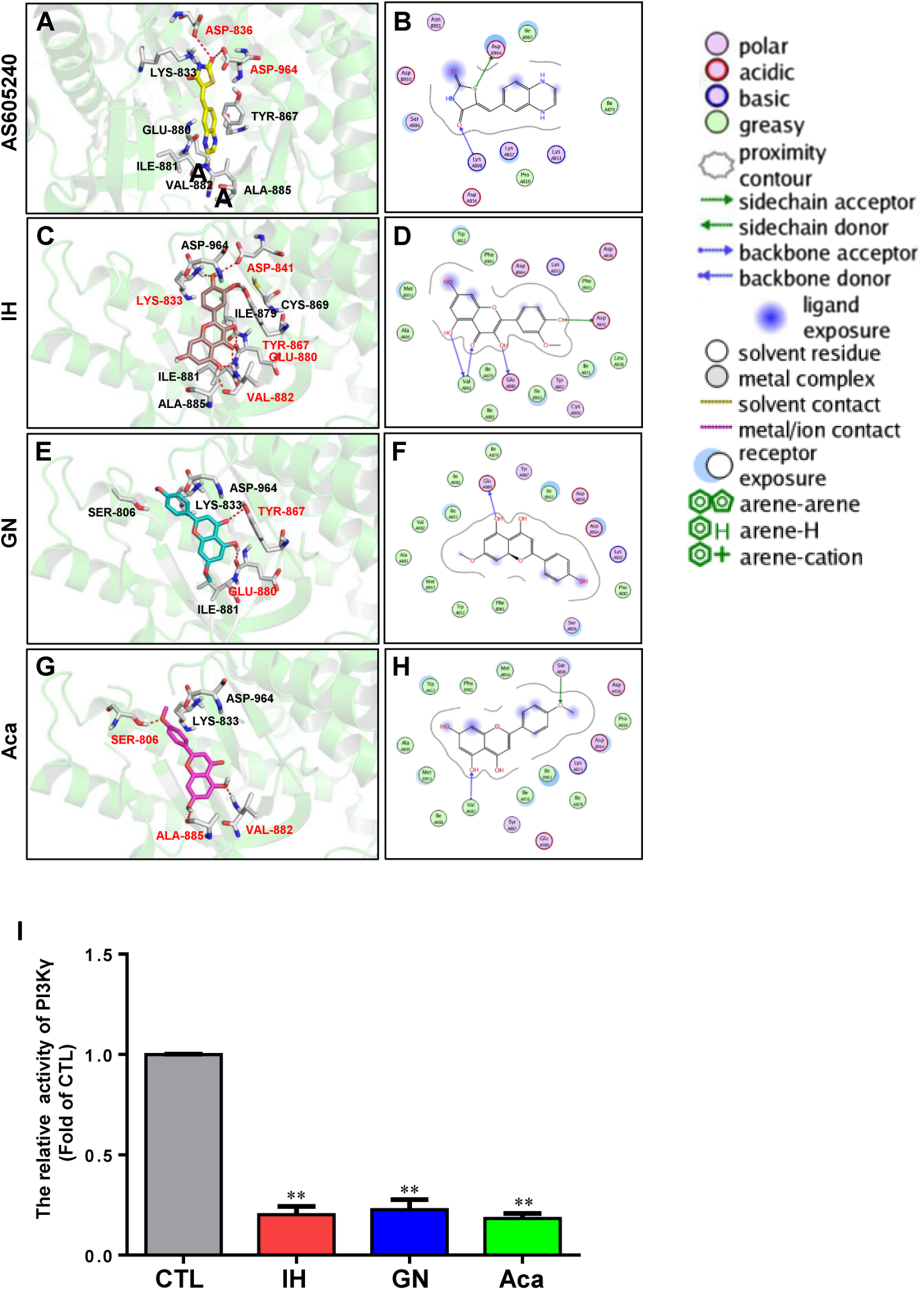
Molecular docking of AS605240, IH, GN and Aca in the ATP binding pocket of PI3Kγ (PDB: 2A5U). (**A**–**G**) PI3Kγ’s ATP binding domain crystal structure with AS605240, IH, GN and Aca. (**B**–**H**) The interaction of AS605240, IH, GN and Aca with protein by 2D for the PI3Kγ’s ATP binding domain crystal structure. (**I**) The relative catalytic activity of PI3Kγ from three independent experiments. (mean ± SD, ^**^P < 0.01 vs. the control).

The interaction between PI3Kγ specific inhibitor AS605240 and PI3Kγ in the docking model is almost the same as that in crystal structure (PDBID: 2A5U)^23^ (Fig. 6A,B), showing that AS605240 formed an interaction with the catalytic lysine (LYS833) within the ATP-binding pocket. As well, other residues, such as ASP-836, ASP-964, TYR-867, GLU-880, ILE-881, ALA-885, and VAL-882 played important roles for their binding interaction.

Three active flavonoids (IH, GN, and Aca) were also successfully docked into the ATP-binding pocket of PI3Kγ crystal structure. In the PI3Kγ-IH docking model, IH interacted with PI3Kγ by formation of hydrogen bonds with LYS-833, VAL-882, ASP-841, TYR-867, and GLU-880. Moreover, there are hydrophobic interactions of IH with ASP-964, CYS-869, ILE-879, ILE-881, and ALA-885 (Fig. 6C,D). More hydrogen bonds were found in the PI3Kγ-GN docking model with the side chains of TYR-867 and GLU-880. Other residues as LYS-833, ASP-964, SER-806, and ILE-881 showed hydrophobic interaction together with GN (Fig. 6E,F). Similarly, Aca interacted with PI3Kγ by formation of hydrogen bonds with SER-806, ALA-885, and VAL-882. In addition, the hydrophobic interaction of acacetin with LYS-833 and ASP-964 was observed in the PI3Kγ-Aca docking model (Fig. 6G,H). These docking results indicated that these flavonoids are able to bind with PI3Kγ by interaction with the important residues for the catalytic activity, such as LYS-833 and ASP-964, which are similar to that of the known PI3Kγ inhibitor AS605240. By using PI3K kinase assay, we found that treatment of breast cancer MDA-MB-231 cells with IH, GN, and Aca resulted in significant decreases in activities of PI3Kγ (Fig. 6I), further supporting the docking prediction.

### IH, GN, and Aca inhibit PI3K/AKT/mTOR/p70S6K/ULK1 signaling pathway via reducing PI3Kγ-p110 expression

**Figure 7:**
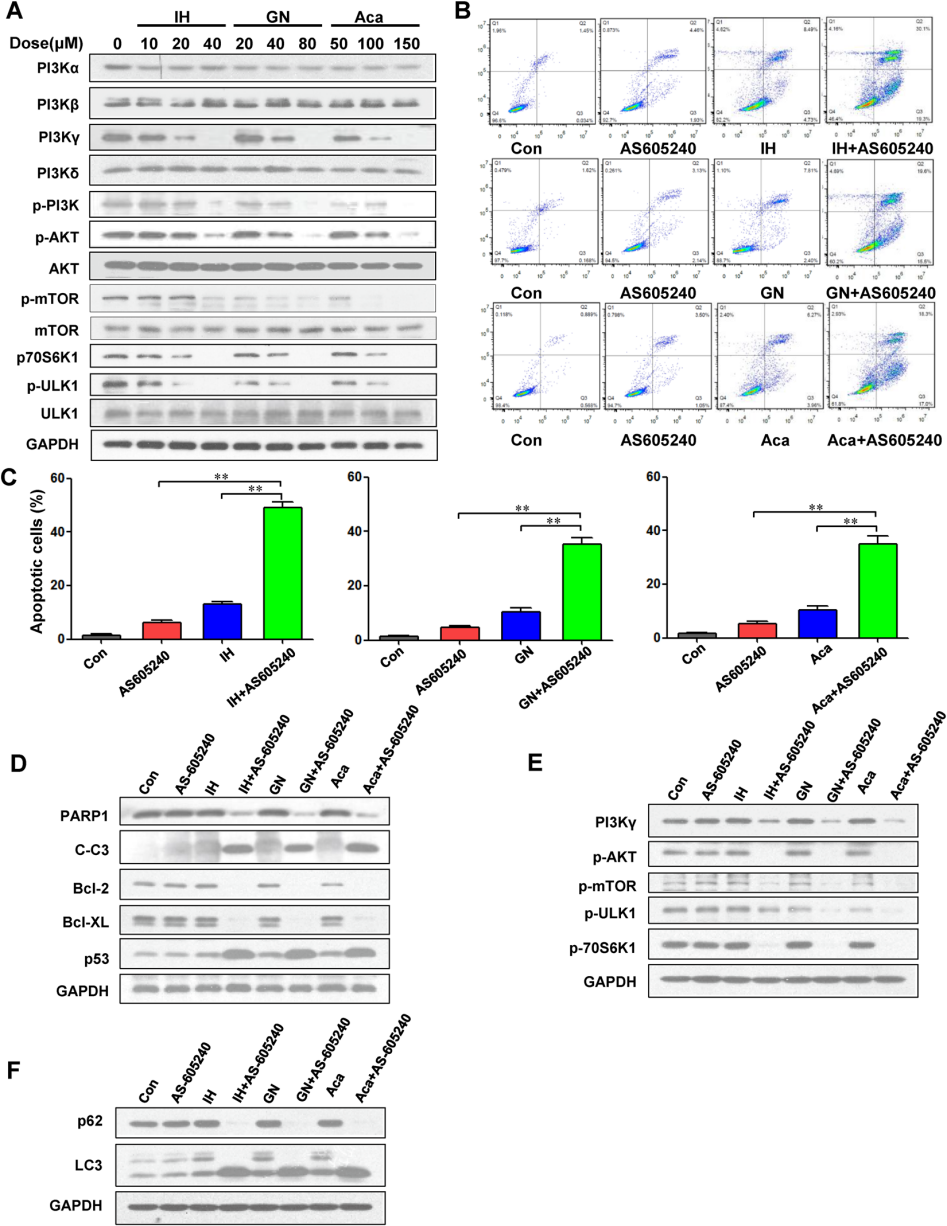
IH, GN and Aca inhibit PI3K/AKT/mTOR/p70S6K/ULK1 signaling pathway via reducing PI3Kγ-p110 expression. (**A**)MDA-MB-231 cells were treated with IH, GN, Aca at indicated concentrations for 24 h, cell lysates were prepared and subjected to western blot using antibodies against PI3Kα, PI3Kβ, PI3Kγ, PI3Kδ, p-PI3K, p-AKT, AKT, p-mTOR, mTOR, p-p70S6K, p-ULK and ULK1. (**B**,**C**) MDA-MB-231 cells were pretreated with 20 μM AS605240 for 2 h, followed by treatment with or without 20 μM IH, 20 μM GN and 50 μM Aca for 48 h. Cells were stained with Annexin V/PI, and the percentage of apoptotic cells were showed as mean ± S.D. for three independent experiments (**P < 0.01). (**D**–**F**) Cells were treated as B and C, cell lysates were prepared and subjected to western blot analysis using antibodies against PARP, cleaved-caspase 3 (C-Caspase 3), Bcl-2, Bcl-xL, p53, PI3Kγ, p-Akt, p-mTOR, p-ULK p70S6K, p62 and LC3.

To further confirm whether PI3Kγ is involved in three flavonoids-mediated inhibition of cell proliferation and induction of apoptosis and autophagy, western blot analysis was employed. Exposure of MDA-MB-231 cells to IH, GN, and Aca resulted in dose-dependent decreases in levels of PI3Kγ-p110. In contrast, these flavonoids had little or no effect on expression of PI3Kα, PI3Kβ, and PI3Kδ (Figs 7A, S8A). In addition, these flavonoids had little or no effect on levels of p-ERK, p-p38, and p-JNK (data not shown). It is well known that AKT, mTOR, p70S6K, and ULK are PI3K downstream molecules which regulate cell proliferation, apoptosis and autophagy^24^. We next examined the effects of three flavonoids on expression of p-AKT, p-mTOR, p-p70S6K, and p-ULK by using western blot analysis. Treatment of MDA-MB-231 cells with IH, GN, and Aca led to decreases in levels of p-AKT, p-mTOR, p-p70S6K, and p-ULK in a dose-dependent manner (Figs 7A, S8A). These events were also confirmed in MCF-7 cells-treated with IH, GN, and Aca (Fig. S9A and S9B). Such findings indicate that inactivation of PI3K/AKT/mTOR/p70S6K/ULK1 signaling pathway could play a crucial role in inhibition of cell proliferation and induction of apoptosis and autophagy mediated by flavonoids via reducing the expression of PI3Kγ.

### Inhibition of PI3Kγ activity by AS605240 potentiates IH, GN, and Aca-induced apoptosis

The preceding findings implied that inactivation of PI3Kγ might play an important role in flavonoids-mediated lethality. To test this possibility, cells were coexposed to three flavonoids and the PI3Kγ specific inhibitor AS605240, and apoptosis was monitored. Coadministration of a nontoxic concentration of AS605240 (i.e. 20 μM; 6.31%) with a modestly toxic concentration of IH (20 μM; 13.21%), GN (20 μM; 10.39%) and Aca (50 μM; 10.27%) resulted in a pronounced increase in apoptosis (i.e. to 49.22%, 35.43%, and 35.14% respectively) (Fig. 7B,C). Western blot analysis revealed that coadministration of AS605240 and three flavonoids at concentrations that were ineffective or marginally effective by themselves resulted in pronounced increase in activation of caspases-3 and degradation of PARP1 (Figs 7D, S8B). Combined treatment also resulted in potentiation of Bcl-2 and Bcl-xL downregulation, and p53 upregulation (Figs 7D, S8B). In addition, coadministration of AS605240 and three flavonoids resulted in the virtual abrogation of Akt, mTOR, p70S6K, and ULK activations (Fig. 7E,F, Fig. S8C and S8D), Together, these findings suggest that downregulation of PI3Kγ plays a critical role in regulating the lethality of three flavonoids in human breast cancer cells.

## Discussion

The ability of flavonoids to inhibit cell proliferation and induce apoptosis or autophagy in human cancer cells has stimulated intense interest in their potential as anti-cancer agents. The present results indicate that three flavonoids (IH, GN, and Aca) isolated from *T. kirilowii* exhibited anti-cancer activities (i.e. inhibition of cell proliferation by cell cycle arrest at G2/M phase, induction of apoptosis and autophagy). Our results also provide detailed molecular mechanistic information as to how three flavonoids exert their anti-cancer activities on human breast cancer cells (i.e. by inactivation of PI3K, Akt, mTOR, p70S6K, and ULK, upregulation of p53, and downregulation of Bcl-2 and Bcl-xL).

Previous studies indicated that IH suppressed cell proliferation through cell cycle arrest at G2/M phase in human colon cancer cells^25^. GN inhibited cell proliferation via cell cycle arrest at S phase in mouse melanoma B16F10 cells^26^. Aca inhibited cell proliferation via cell cycle arrest at G1 phase in human non-small cell lung cancer A549 cells or via cell cycle arrest at G2/M phase in human prostate cancer DU145 cells^27,28^. Our studies indicated that IH, GN, and Aca inhibited cell proliferation through cell cycle arrest at G2/M phase in human breast cancer MDA-MB-231 cells. Cell cycle progression through G2-M is regulated by activation of a complex consisting of catalytic subunit Cdc2 and regulatory subunit cyclin B1 that controls the entry into mitosis^29^. Consistent with these reports, treating breast cancer cells with IH, GN, and Aca decreased the levels of phospho-Cdc2 and cyclin B1, suggesting that dephosphorylation of Cdc2 and downregulation of cyclin B1 could be the underlying molecular events contributing to G2/M arrest caused by these flavonoids in breast cancer cells.

Increasing evidence reveal that cell cycle arrests are often followed by or associated with apoptotic death of cancer cells by many cancer therapeutic agents^30^. Our data show that growth inhibitory effect of these flavonoids was accompanied by an induction of apoptosis, which was accompanied by activation of caspase-3 and degradation of PARP1. We also observed that treatment of G2/M-synchronized cells (by nocodazole) with these flavonoids resulted in a pronounced increase in the proportion of sub-G1 cell population and apoptosis. Such findings suggest that these flavonoids inhibit cell proliferation via cell cycle at G2/M phase, and culminating in apoptosis in human breast cancer cells.

The tumor suppressor gene p53 has been shown to regulate the cell cycle at the G2/M checkpoint in response to DNA damage^30^. Upregulation of p53 leads to G2/M arrest through the induction of CDK inhibitor p21, which inhibits cdc2 activity by binding directly to Cdc/Cyclin B1 complexes. p53 has also been shown to regulate apoptosis through inhibiting the expression of anti-apoptotic proteins (i.e. Bcl-2, Blc-xL, Mcl-1, etc.)^21^. Consistent with these reports, our data reveal that p53 plays an important role in these active flavonoids-mediated cell cycle arrest at G2/M phase and apoptosis based on the following results: (i) treatment of breast cancer cells with these flavonoids increased the expression of p53; (ii) these flavonoids induced cell cycle arrest at G2/M phase through inhibiting the expression/activity of p53′s downstream molecules (i.e. activity of Cdc2 and expression of cyclin B1); (iii) these flavonoid compounds induced apoptosis through downregulation of p53′s downstream molecules (i.e. Bcl-2 and Bcl-xL). These findings suggest that upregulation of p53 by these flavonoids not only caused cell cycle arrest at G2/M phase, but also triggered apoptosis in breast cancer cells.

Autophagy is a catabolic process that enables the sequestration and lysosomal degradation of cytoplasmic organelles and proteins and that is important for the maintenance of genomic stability and cell survival^31^. Current cancer therapies including chemotherapy and radiation are known to induce autophagy within tumor cells^21^. Accordingly, small-molecules that induce autophagy may have broad therapeutics applications for cancer treatment. Recently, many flavonoids have been found to exhibit anti-cancer effects through the modulation of autophagy. For example, plant flavonoid wogonin has been shown to induce cancer cell death through inhibition of autophagy^32^. In contrast, many flavonoids have been shown to induce cell death through induction of autophagy. For example, curcumin is able to induce both autophagy and apoptosis in chronic myeloid leukemia cells via downregulation of the Bcl-2 protein^33^. Quercetin induces extensive autophagy in epithelial cancer cells, which led to cell cycle arrest and induction of apoptosis^34^, etc. Our results indicate that IH, GN, and Aca could be autophagic inducers that induce autophagosome accumulation in breast cancer cells based on the following findings: First, treating cells with these flavonoids resulted in a marked increase in EGFP-LC3 puncta formation and accumulation of LC3B-II in breast cancer cells. Second, treatment with these flavonoids decreased the levels of p62, which is a protein that acts as a cargo receptor for the autophagic degradation of substrates and is an indicator of autophagy induction. Third, treatment with these flavonoids increased the levels of ATG5, which is considered to be essential molecule for the induction of autophagy. Fourth, by assessment of EGFP-LC3 and mRFP-LC3 puncta colocalization, we found that cells exposed to these flavonoids led to the production of large amounts of red-only puncta, which is similar to that in cells treated with rapamycin, a typical autophagy inducer. Therefore, our findings suggest that ATG5 participates in these flavonoids-induced autophagosome accumulation.

Phosphoinositide 3-kinase (PI3K) activity is stimulated by diverse oncogenes and growth factor receptors, and elevated PI3K signaling is considered a hallmark of cancer and is a key therapeutic target in the treatment of cancer^35^. Class I PI3Ks are of particular therapeutic interest, given their importance in the development of human cancers. Class I PI3Ks are heterodimers, comprising a catalytic (p110α, p110β, p110δ, or p110γ) and a regulatory (p85α, p55α, p50α, p85β, p55γ, or p101) subunit. The activated p110 subunit then activates protein kinase B/AKT and downstream effectors including mTOR^36^. The PI3K/Akt/mTOR/p70S6K pathway is likely the most frequently activated pathway in human cancers, making it an attractive target for anti-cancer drug development. Inhibition of signaling along this pathway can result in both decreased cellular proliferation and promotion of cellular death (including apoptosis and autophagy)^37^. Increasing evidence indicate that several flavonoids (i.e. procyanidins, luteoloside, and hesperidin) induce apoptosis and/or autophagy through inhibition of PI3K/Akt/mTOR/p70S6K pathway^38–40^. Based on our results, we speculate that interruption of PI3K/AKT/mTOR/p70S6K signaling pathway may contribute to these flavonoids-induced cell cycle arrest at G2/M phase, apoptosis, and autophagy through reducing PI3Kγ-p110 expression. First, molecular docking indicated that these flavonoids are able to bind with PI3Kγ by interaction with the important resides for the catalytic activity, such as LYS-833 and ASP-964, which are similar to that of the known PI3Kγ inhibitor AS605240. Second, treatment of cells with these flavonoids decreased the expression of PI3Kγ, but did not affect the expression of other PI3K isoforms including PI3Kα, β, and δ. Third, pretreatment of cells with PI3Kγ specific inhibitor AS605240 significantly enhanced these flavonoids-mediated reduction of PI3Kγ and inactivation of AKT, mTOR, and p70S6K. Fourth, pretreatment of cells with PI3Kγ specific inhibitor AS605240 significantly enhanced these flavonoids-mediated apoptosis. Thus, our finding suggest that these flavonoids-mediated cell cycle arrest at G2/M phase, apoptosis, and autophagy might be attributed to PI3Kγ-p110 downregulation, which leads to interruption of PI3K/AKT/mTOR/p70S6K/ULK signaling pathway.

In conclusion, we isolated and identified, for the first time, eight flavonoids from *T. kirilowii*. Three flavonoids (IH, GN, and Aca) induced cell cycle arrest at G2/M phase, apoptosis, and autophagy in human breast cancer cells, in which downregulation of PI3Kγ-p110 and consequent interruption of PI3K/AKT/mTOR/p70S6K/ULK signaling pathway might be closely involved. Our studies provide novel insights into the anticancer activities of selected active flavonoids and their potential uses in anticancer therapy.

## Electronic supplementary material

Dataset 1
