# Supplementary material for: Flavonoids inhibit cell proliferation and induce apoptosis and autophagy through downregulation of PI3Kγ mediated PI3K/AKT/mTOR/p70S6K/ULK signaling pathway in human breast cancer cells
Source: Sci Rep. 2018 Jul 26;8:11255. doi: 10.1038/s41598-018-29308-7 (PMC6062549; doi:10.1038/s41598-018-29308-7)
Supplement: Supplementary file 1 — Dataset 1 [file 41598_2018_29308_MOESM1_ESM.docx]

**Flavonoids inhibit cell proliferation and induce apoptosis and autophagy through downregulation of PI3Kγ mediated PI3K/AKT/mTOR/p70S6K/ULK signaling pathway in human breast cancer cells**

Hong-Wei Zhang^1^, Jin-Jiao Hu^1^, Ruo-Qiu Fu, Xin Liu, Yan-Hao Zhang, Jing Li, Lei Liu, Yu-Nong Li, Qin Deng, Qing-Song Luo, Qin Ouyang, Ning Gao^*^

**Authors’ Affiliations**:

College of Pharmacy, 3rd Military Medical University, Chongqing 400038, China

^1^These authors contributed equally to this work

^*^**Corresponding author.** Email address: [gaoning59@163.com](mailto:gaoning59@163.com)


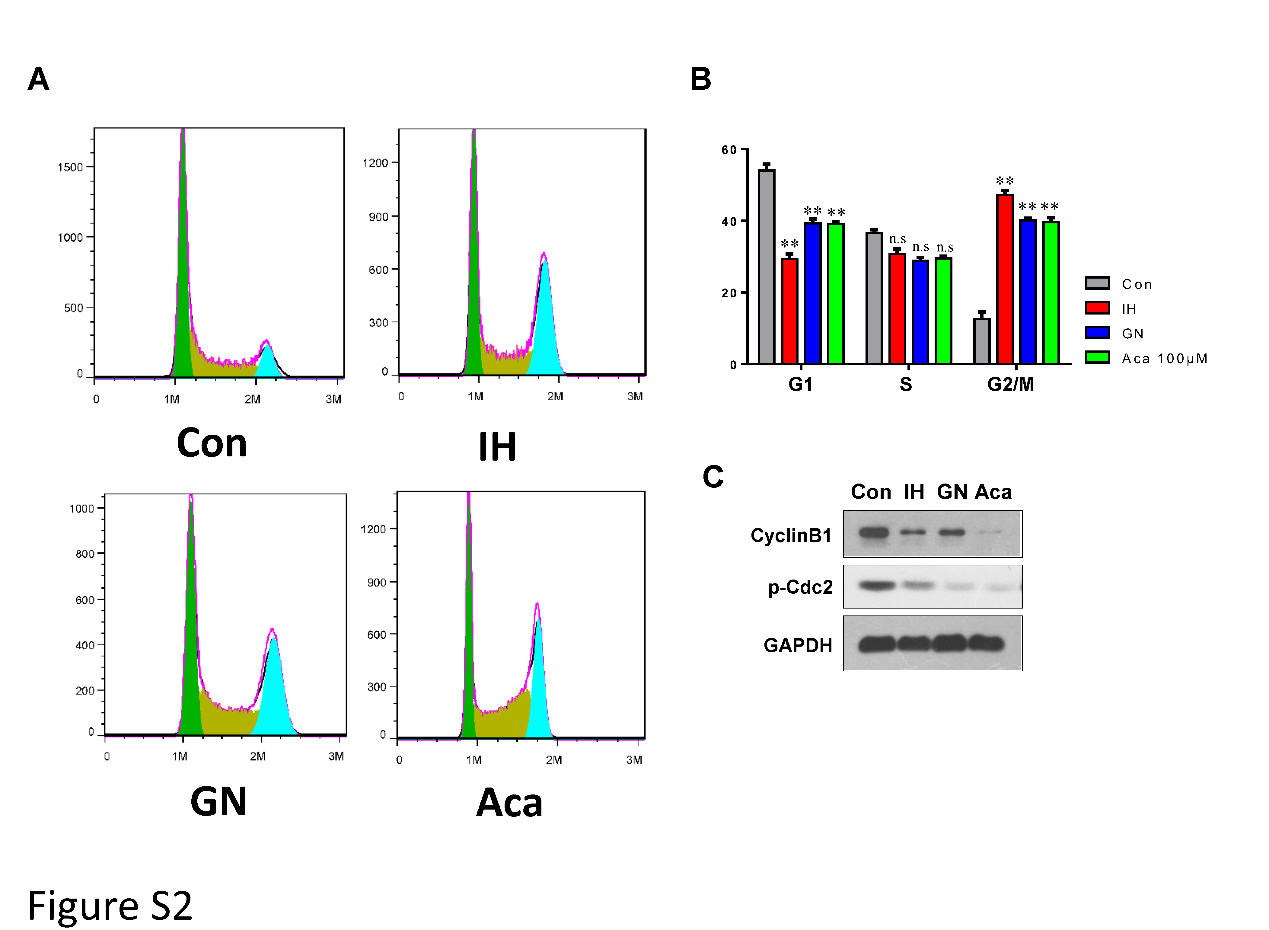


**Figure S1.**

MCF-7 cells were treated with 40 μM IH, 80 μM GN and 150 μM Aca for 24 h, (A, B) Cells were stained with cell cycle staining solution and analyzed using a flow cytometer. The percentage of cells in each phase is showed as mean ± S.D. for three independent experiments (^n.s^ *P* >0.05, ^*^*P*<0.05 or ^**^*P*<0.01 vs. the control). (C) Cell lysates were prepared and analyzed by western blotting using antibodies against phospho-Cdc2 and cyclin B1.


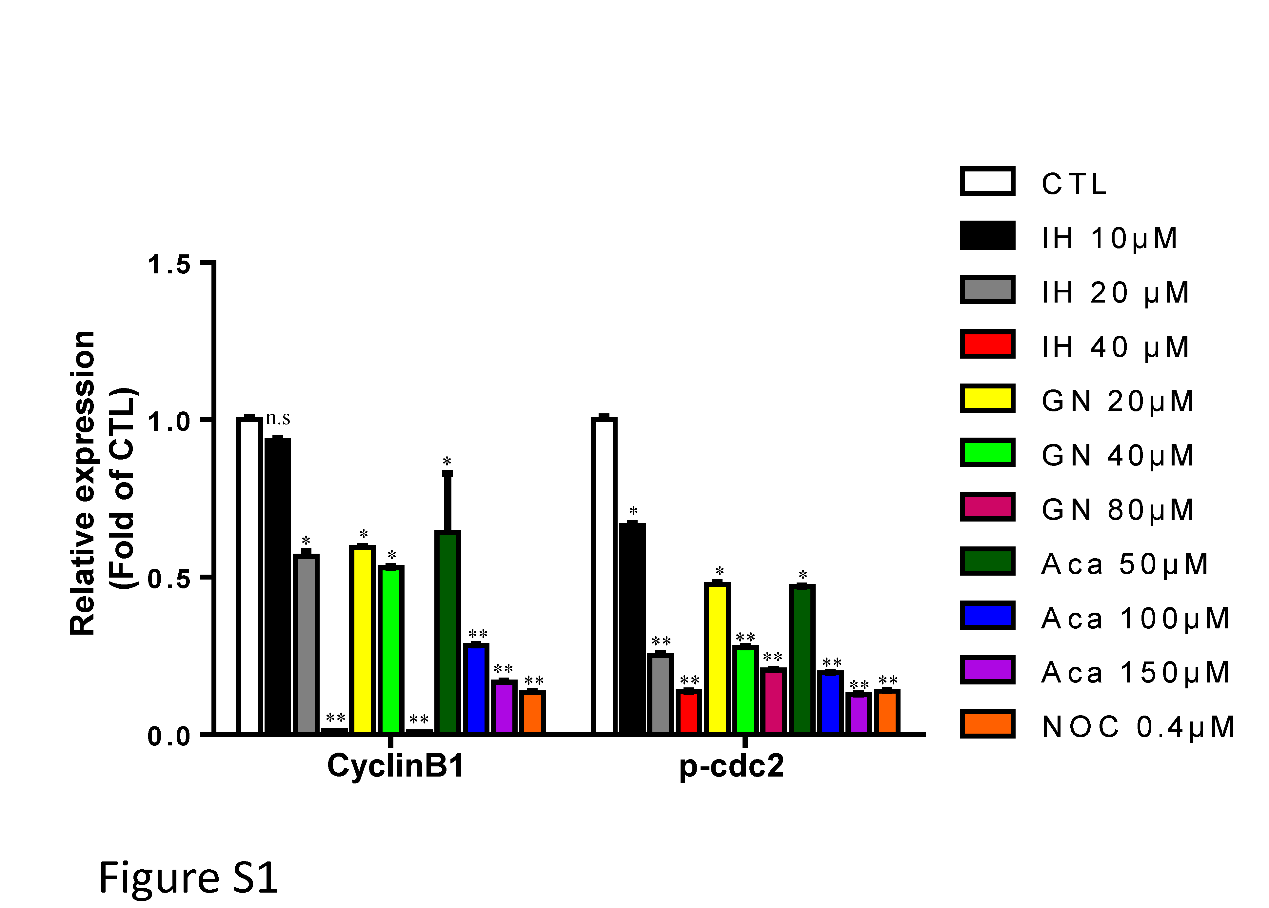


**Figure S2.**

The relative levels of CyclinB1 and p-Cdc2 were quantified by densitometry and normalized to GAPDH in three independent experiments. (mean ± SD, ^n.s^*P*>0.05,^*^*P*<0.05 or ^**^*P*<0.01 vs. the control).

**
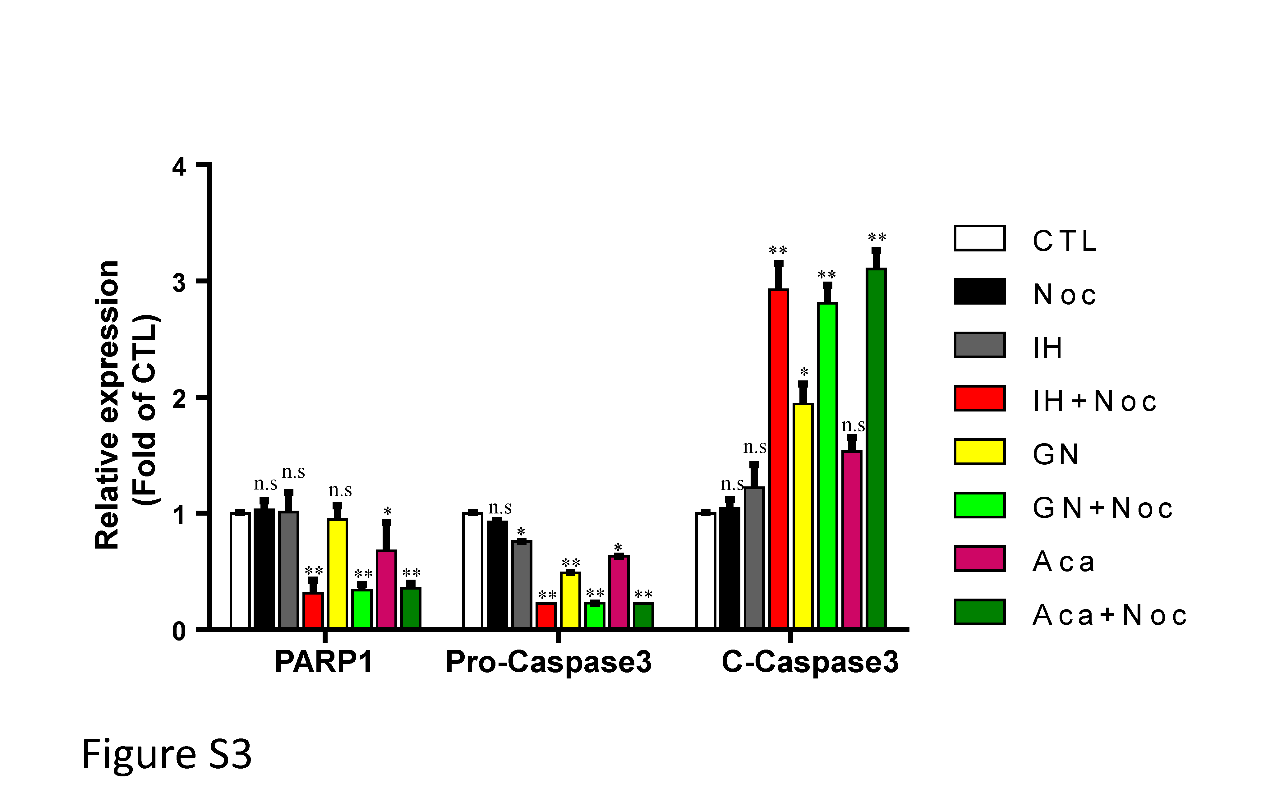
**

**Figure S3**

The relative levels of PARP1, pro-caspase3 and C-Caspase3 were quantified by densitometry and normalized to GAPDH in independent experiments. (mean ± SD, ^n.s^*P*>0.05,^*^*P*<0.05 or ^**^*P*<0.01 vs. the control).


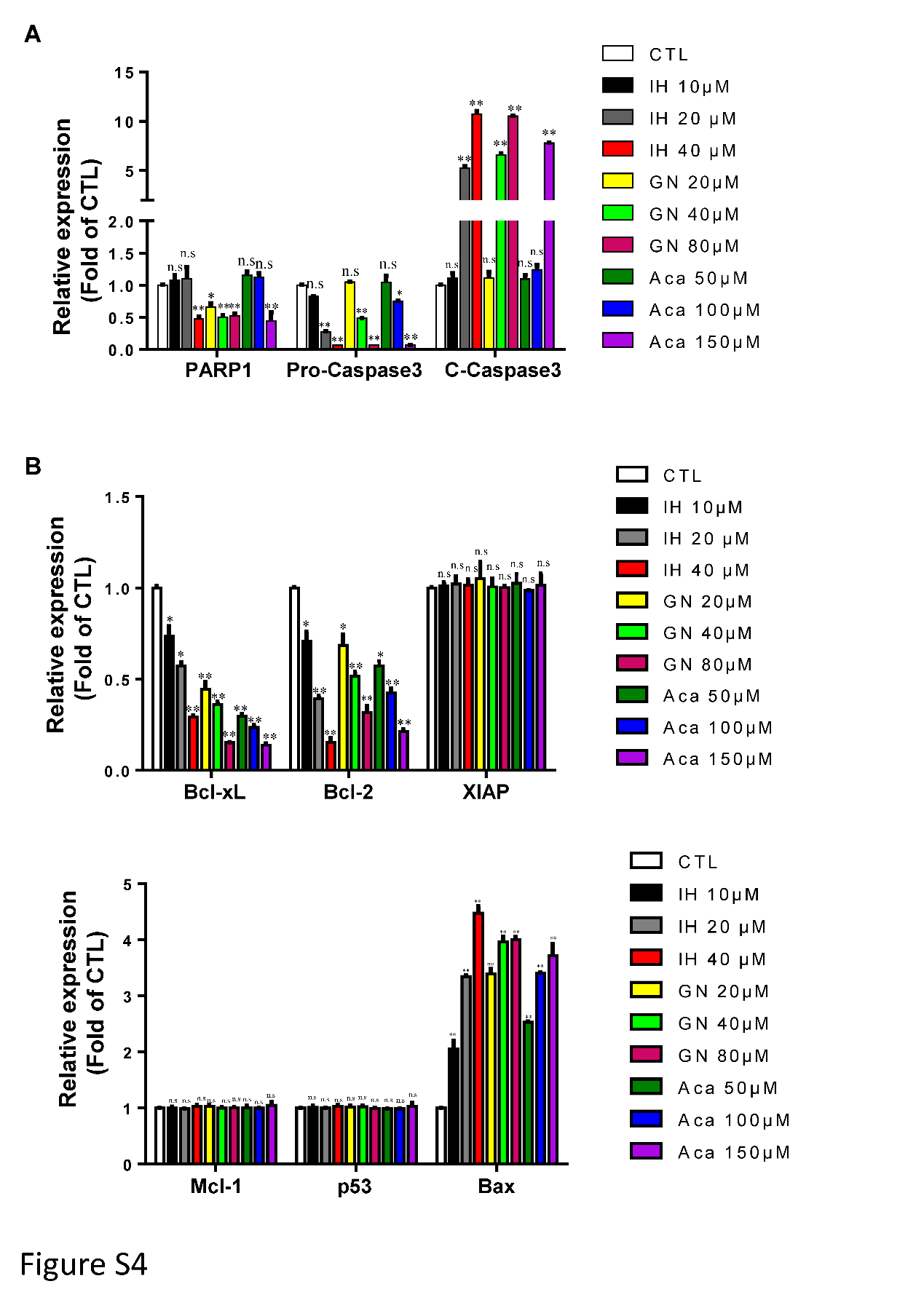


**Figure S4.**

(A, B) The relative levels of PARP1, pro-caspase3, C-caspase 3, Bcl-2, Bcl-xL, p53, Bax, XIAP and Mcl-1 were quantified by densitometry and normalized to GAPDH in three independent experiments. (mean ± SD, ^n.s^*P*>0.05,^*^*P*<0.05 or ^**^*P*<0.01 vs. the control).


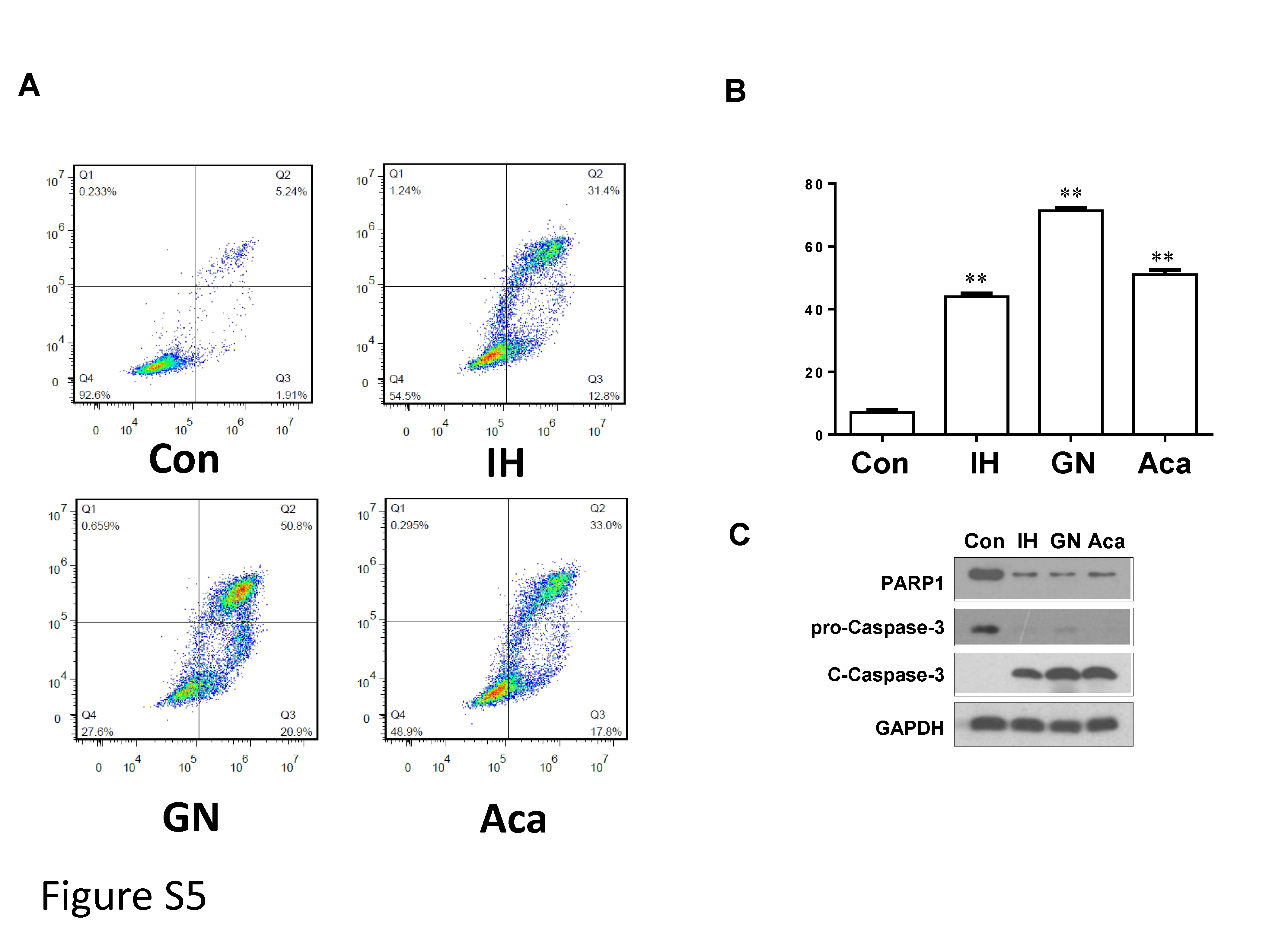


**Figure S5**

MCF-7 cells were treated with 40 μM IH, 80 μM GN and 150 μM Aca for 48 h, (A, B) The percentage of apoptotic cells were determined by flow cytometry using Annexin V/PI staining for three independent experiments. (mean±SD, ^n.s^*P* > 0.05,^*^ *P* < 0.05 or ^**^*P* < 0.01 vs. the control). (C) Cell lysates were prepared and analyzed by western blotting using antibodies against PARP1, pro-caspase-3, cleaved-caspase 3 (C-Caspase 3).


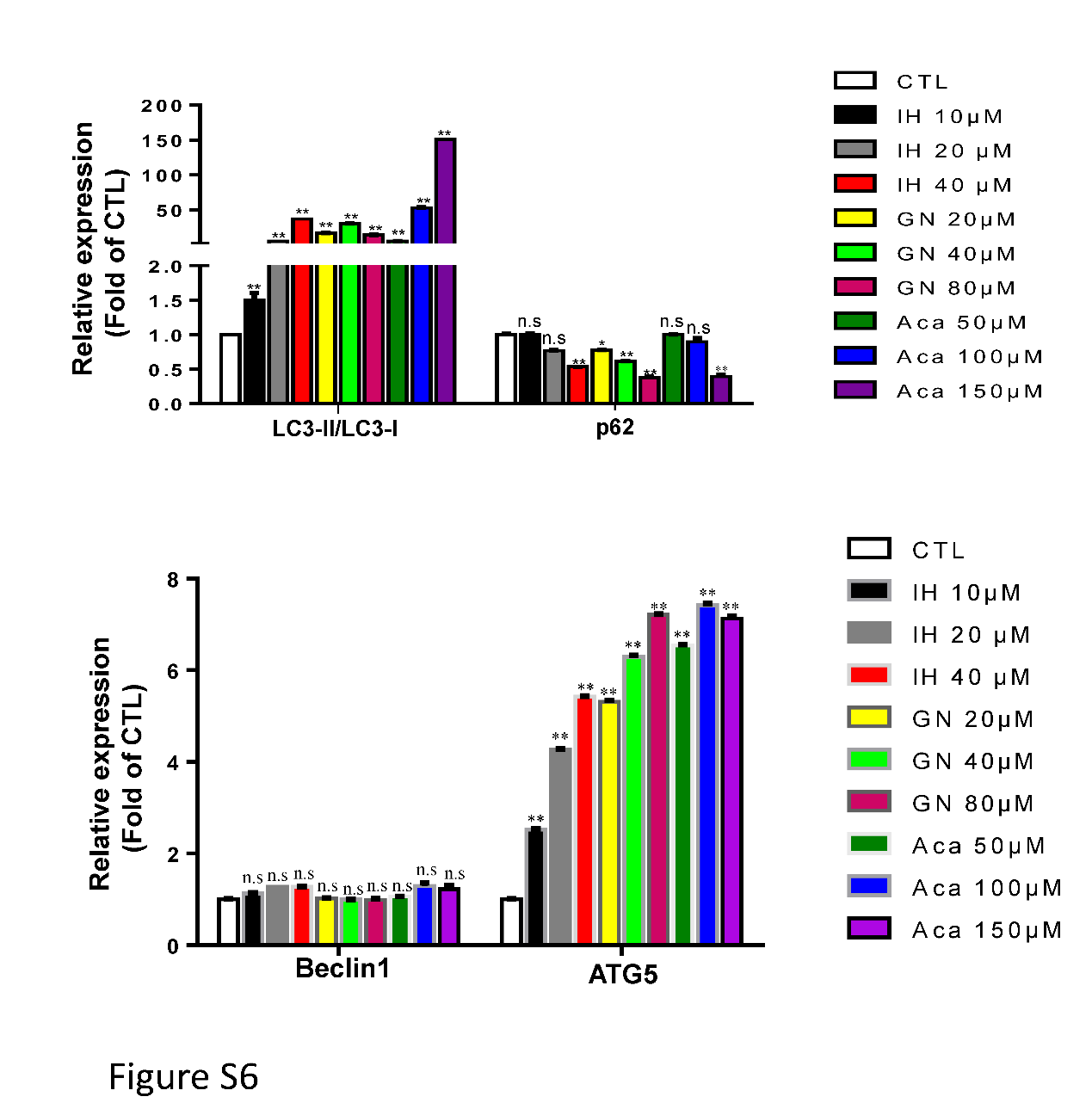


**Figure S6**

The relative levels of LC3-II/LC3-I, p62, Beclin1 and ATG5 were quantified by densitometry and normalized to GAPDH in three independent experiments. (mean ± SD, ^n.s^*P*>0.05,^*^*P*<0.05 or ^**^*P*<0.01 vs. the control).


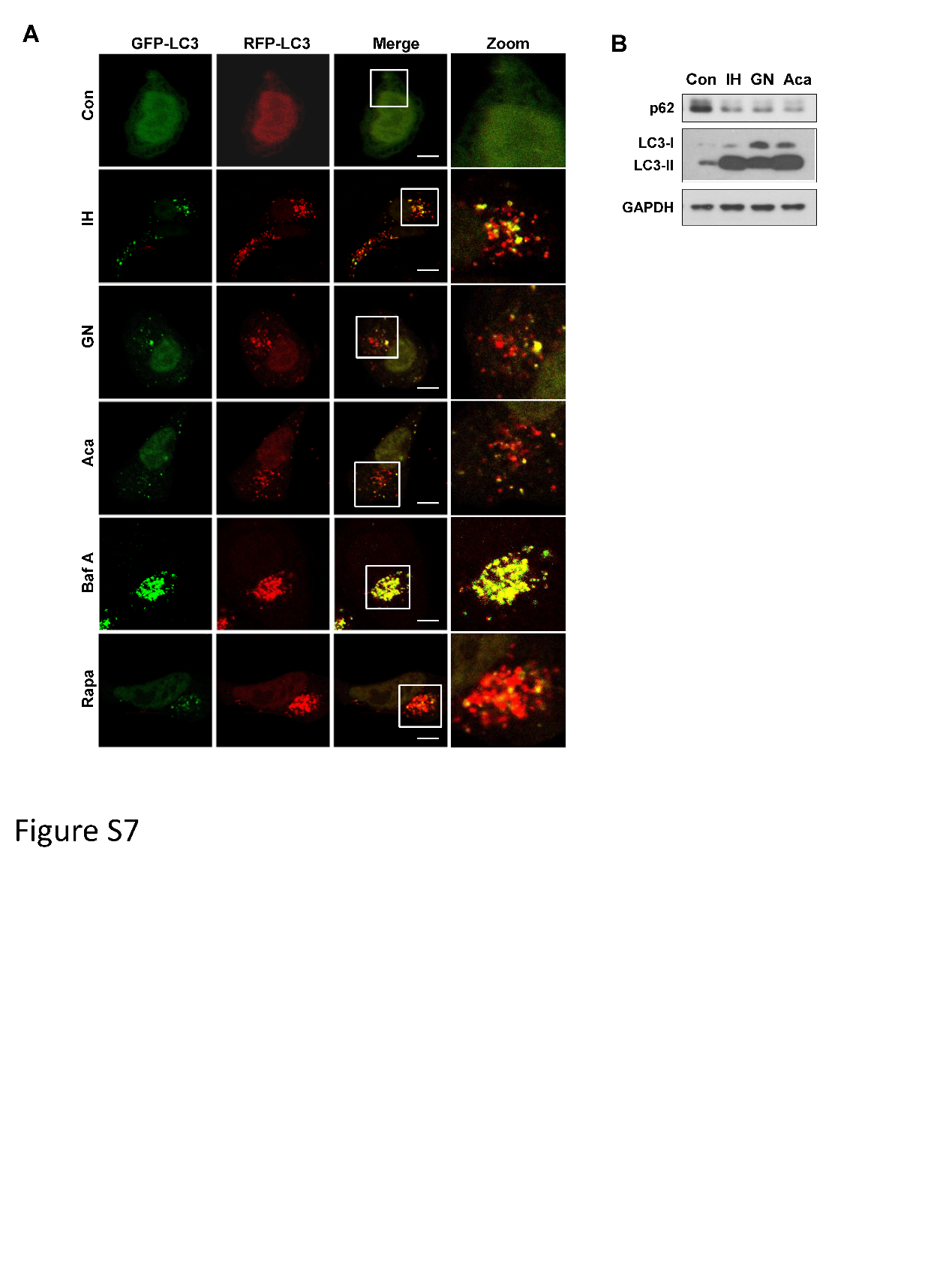


**Figure S7**

1. MCF7 Cells were transfected with a tandem reporter construct (tfLC3), and were exposed to 30 μM IH, 20 μM GN, 100 μM Aca, 20 nM Baf and 0.25 μM Rapa as indicated. The colocalization of EGFP and mRFP-LC3 puncta was examined by confocal microscopy. Scale bars: 10 μm. (B) Cells were exposed to indicated concentrations of IH, GN and Aca for 24 h, the expression of autophagy-related proteins (LC3B-II, p62) were detected by western blot analysis.


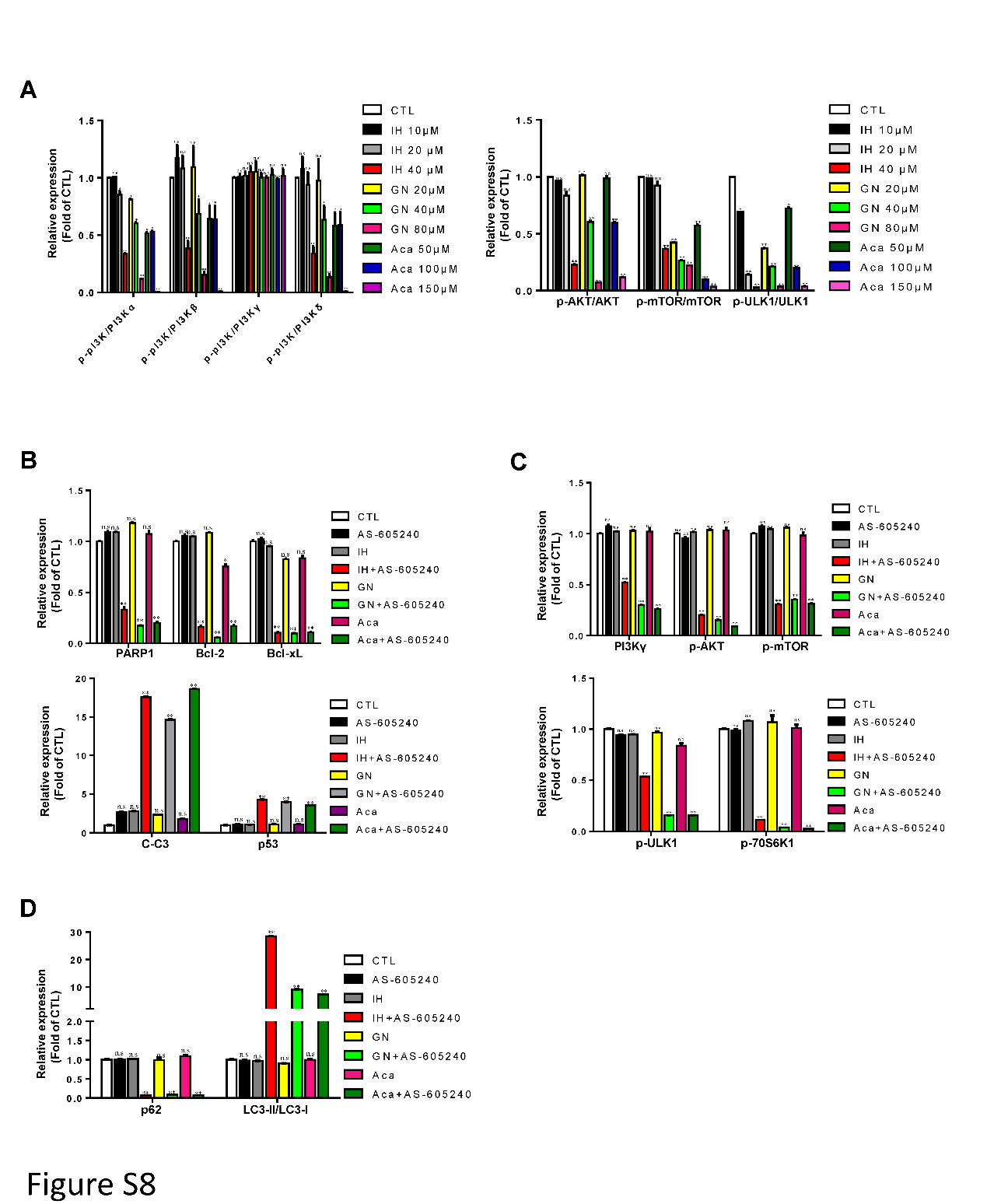


**Figure S8**

1. The ratios of p-PI3K/PI3Kα，p-PI3K/PI3Kβ，p-PI3K/PI3Kγ，p-PI3K/PI3Kδ, p-AKT/AKT, p-mTOR/mTOR and p-ULK1/ULK1 were quantified by densitometric analysis in three independent experiments. (mean ± SD, ^n.s^*P*>0.05,^*^*P*<0.05 or ^**^*P*<0.01 vs. the control). (B, C and D) The relative levels of PARP, C-Caspase 3, Bcl-2, Bcl-xL, p53, PI3Kγ, p-Akt, p-mTOR, p-ULK, p70S6K, p62 and LC3-II/LC3-I were quantified by densitometry and normalized to GAPDH in three independent experiments. (mean ± SD, ^n.s^*P*>0.05,^*^*P*<0.05 or ^**^*P*<0.01 vs. the control).


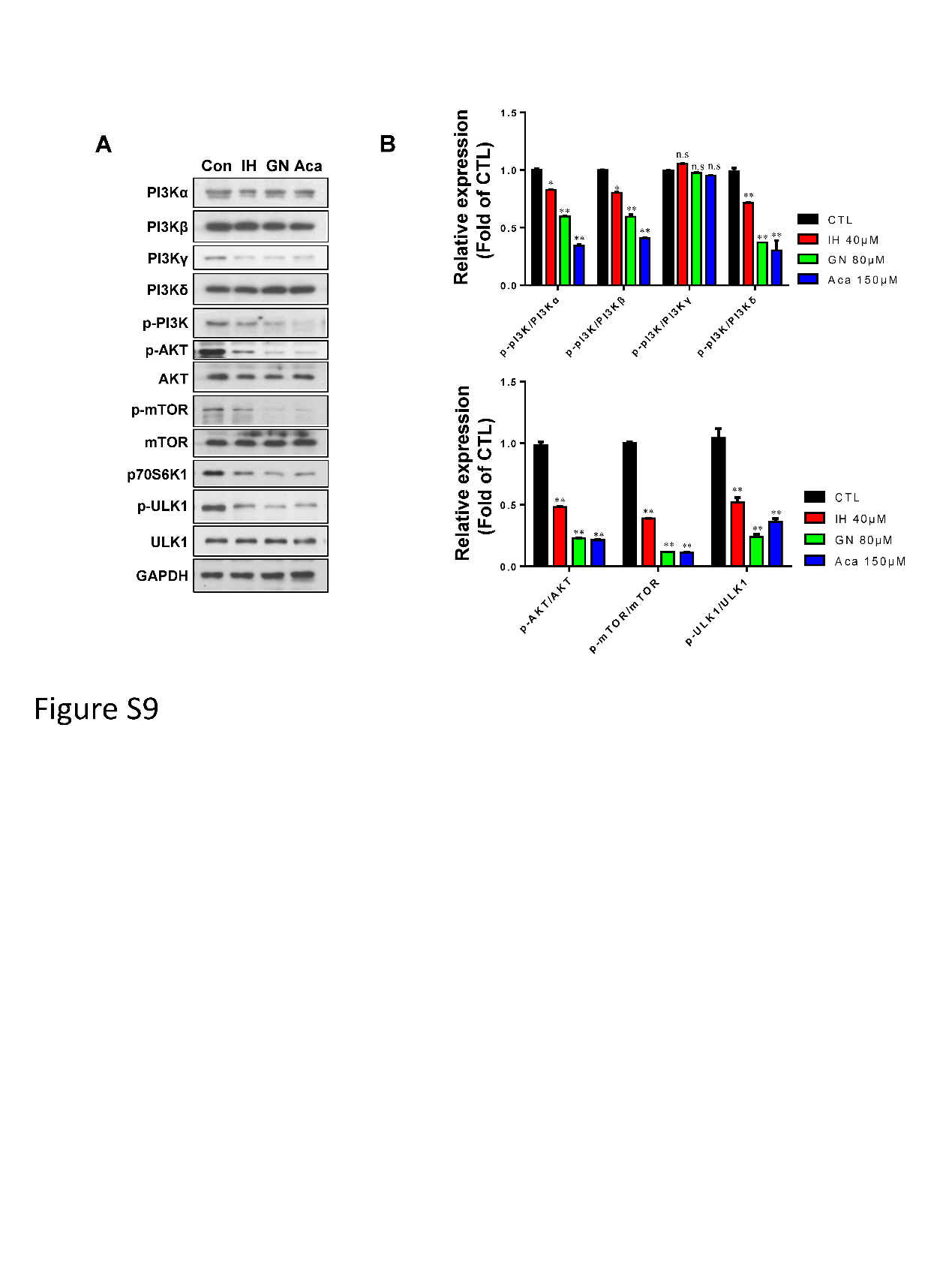


**Figure S9**

1. MCF-7 cells were treated with 40 μM IH, 80 μM GN and 150 μM Aca for 24 h, cell lysates were prepared and subjected to western blot using antibodies against PI3Kα, PI3Kβ, PI3Kγ, PI3Kδ, p-PI3K, p-AKT, AKT, p-mTOR, mTOR, p-p70S6K, p-ULK and ULK1. (B) The ratios of p-PI3K/PI3Kα，p-PI3K/PI3Kβ，p-PI3K/PI3Kγ，p-PI3K/PI3Kδ, p-AKT/AKT, p-mTOR/mTOR and p-ULK1/ULK1 were quantified by densitometric analysis in three independent experiments. (mean ± SD, ^n.s^*P*>0.05,^*^*P*<0.05 or ^**^*P*<0.01 vs. the control).
